# A68 HOW REAL ARE YOUR SURVEY RESPONDENTS? IDENTIFYING FRAUDULENT RESPONDENTS IN ONLINE SURVEYS – A CASE EXAMPLE IN INFLAMMATORY BOWEL DISEASE (IBD)

**DOI:** 10.1093/jcag/gwac036.068

**Published:** 2023-03-07

**Authors:** K V MacDonald, G C Nguyen, K L Barker, M Harris, M J Sewitch, D A Marshall

**Affiliations:** 1 Community Health Sciences, University of Calgary, Calgary; 2 University of Toronto; 3 Mount Sinai Hospital, Toronto; 4 McGill University, Montreal, Canada

## Abstract

**Background:**

Social media and online surveys are commonly used to recruit and collect data from patients and physicians about GI diseases – they are efficient, convenient, and less resource intensive compared to traditional recruitment approaches and paper surveys. However, online data fraud is increasing and difficult to identify. Online data fraud can include intentional duplicate responses/straight-lining/inattention, bots/malicious software, and professional survey takers who provide fraudulent responses to meet study eligibility.

**Purpose:**

1) Illustrate challenges of identifying fraudulent respondents through an algorithm and verification process we developed for our survey in IBD. 2) Demonstrate potential impact of fraudulent respondents on data and results.

**Method:**

Online survey of Canadian adults (>18 years) with IBD about healthcare processes for managing IBD hosted using Qualtrics. Recruitment was done in clinic and online (mailing lists, social media). A $25 giftcard was offered for participation due to low response after 3 months in field, after which a large influx of ‘respondents’ occurred. Most were fraudulent although not obvious at first. To mitigate further fraudulent responses, we added the following to our survey: reCAPTCHA score, repeated question (year of IBD diagnosis), duplicate ID score, fraud score and honeypot question.

Our algorithm to identify fraudulent responses included 13 binary ‘red flag’ variables: age <18 years, year of diagnosis < year of birth, 2 different year of diagnosis, invalid postal code, survey duration <10 minutes, survey duration 10-15 minutes, suspicious comments for open text questions (x2), duplicate email, suspicious email, duplicate ID score ≥30, fraud score ≥30, and failed honeypot question. These variables were used to generate a fraudulent response score (range: 0-13; 13=most likely fraudulent). ‘Respondents’ with scores >3 were categorized as likely fraudulent. Respondents with scores ≤3 were reviewed individually. Respondents flagged as likely real or unsure were emailed and asked to verify their age; those who correctly verified age were considered likely real and included in the final sample.

**Result(s):**

Of the 4334 ‘respondents’ who started the survey, based on fraudulent response score we identified 75% (n=3258) as likely fraudulent, 17% (n=727) as unsure and 8% (n=349) as likely real. After age verification, 76% (n=3297) were considered likely fraudulent, 14% (n=592) remained unsure, 10% (n=442) were considered likely real, and <1% (n=3) were duplicates of likely real respondents.

**Conclusion(s):**

Despite convenience, social media and online surveys can be prone to fraudulent responses, especially when incentives are offered. We developed an algorithm and verification process to identify fraudulent responses using an IBD survey example. Given that only 10% of the full sample was considered likely real, researchers using social media and online surveys should carefully examine data for fraudulent responses and apply strategies to mitigate risks.

**Please acknowledge all funding agencies by checking the applicable boxes below:**

CCC

**Disclosure of Interest:**

None Declared

